# Supplementary material for: Transmission of community- and hospital-acquired SARS-CoV-2 in hospital settings in the UK: A cohort study
Source: PLoS Med. 2021 Oct 12;18(10):e1003816. doi: 10.1371/journal.pmed.1003816 (PMC8509983; doi:10.1371/journal.pmed.1003816)

# Supplementary material S1 Figure

**The timelines of potential nosocomial transmission events and the numbers of infectious patients and healthcare workers**

Weekly average numbers of transmission events and infectious patients and healthcare workers in wards with at least 30 members of staff tested and 20 available patient beds. The top row for each panel of graphs shows the weekly average number of patients who had a positive SARS-CoV-2 PCR test and who were defined to have been infected in the hospital on that ward during the indicated week based on the assumed incubation period of five days. The second and third rows for each panel show the numbers of infectious patients defined as having community-acquired SARS-CoV-2 infections (i.e., no hospitalisation in the 20 days prior to first positive tests), and patients who acquired SARS-CoV-2 in the hospital (i.e., inpatient on the sixth day prior to first positive tests) respectively. For these plots, patients were considered to be infectious for a period of ten days, starting one day after the day of the presumed infection event. The last row shows the number of infectious healthcare workers. Healthcare workers were considered infectious from the day of the infection event until a day before their first positive PCR test or report of COVID-19 related symptoms, whichever was earlier.


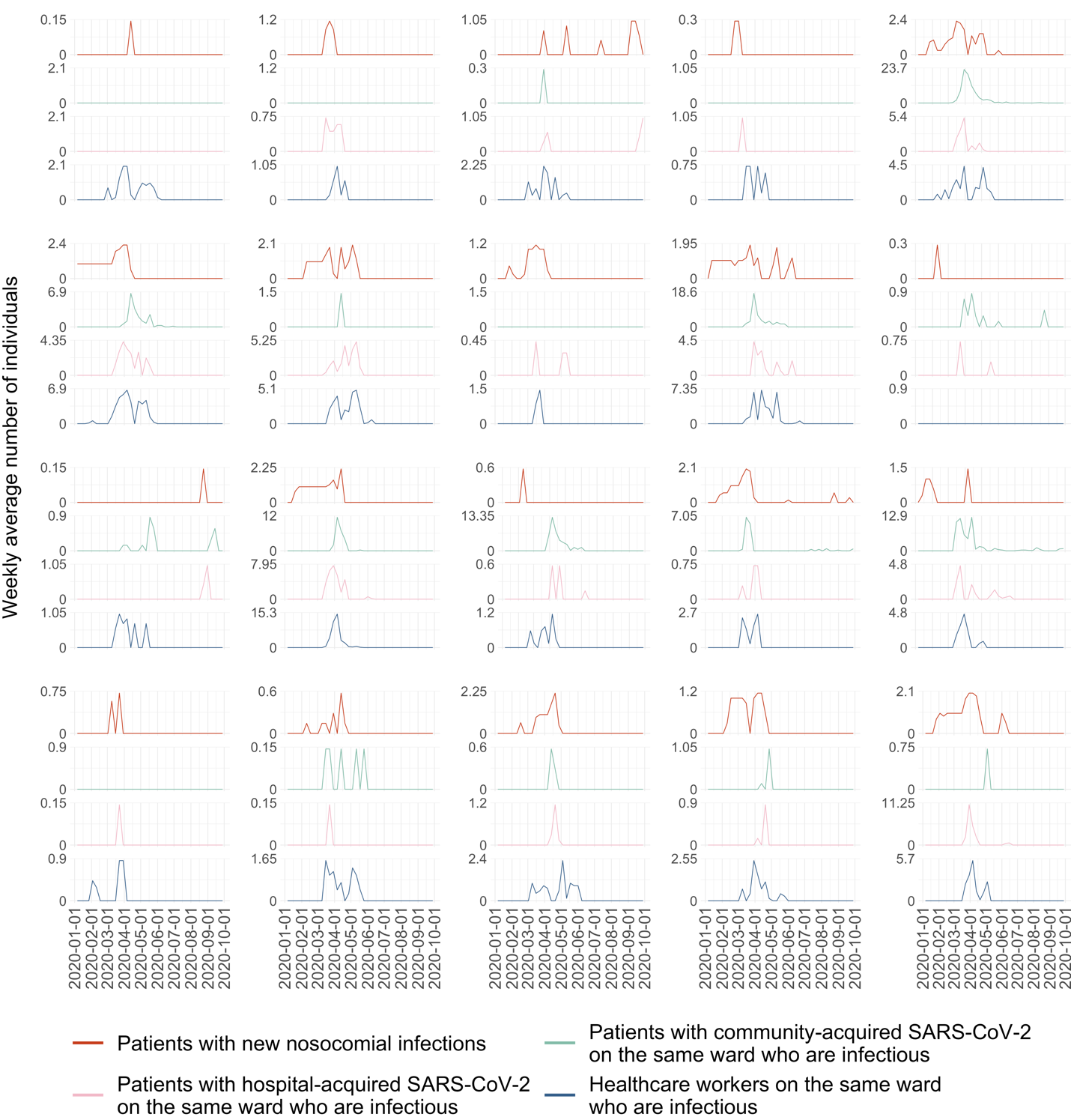

Supplement: S1 Fig — HCW, healthcare worker; SARS-CoV-2, Severe Acute Respiratory Syndrome Coronavirus 2. (DOCX) [file pmed.1003816.s002.docx]
