# Supplementary material for: Transmission of community- and hospital-acquired SARS-CoV-2 in hospital settings in the UK: A cohort study
Source: PLoS Med. 2021 Oct 12;18(10):e1003816. doi: 10.1371/journal.pmed.1003816 (PMC8509983; doi:10.1371/journal.pmed.1003816)
Supplement: S2 Fig — (DOCX) [file pmed.1003816.s003.docx]

# Supplementary material S2 Figure

**Distributions of incubation period and generation time used in the analysis**

Distribution of the incubation periods,[1] generation time[2] (Panel A), and time from symptom onset to onward infection[3] (Panel B).


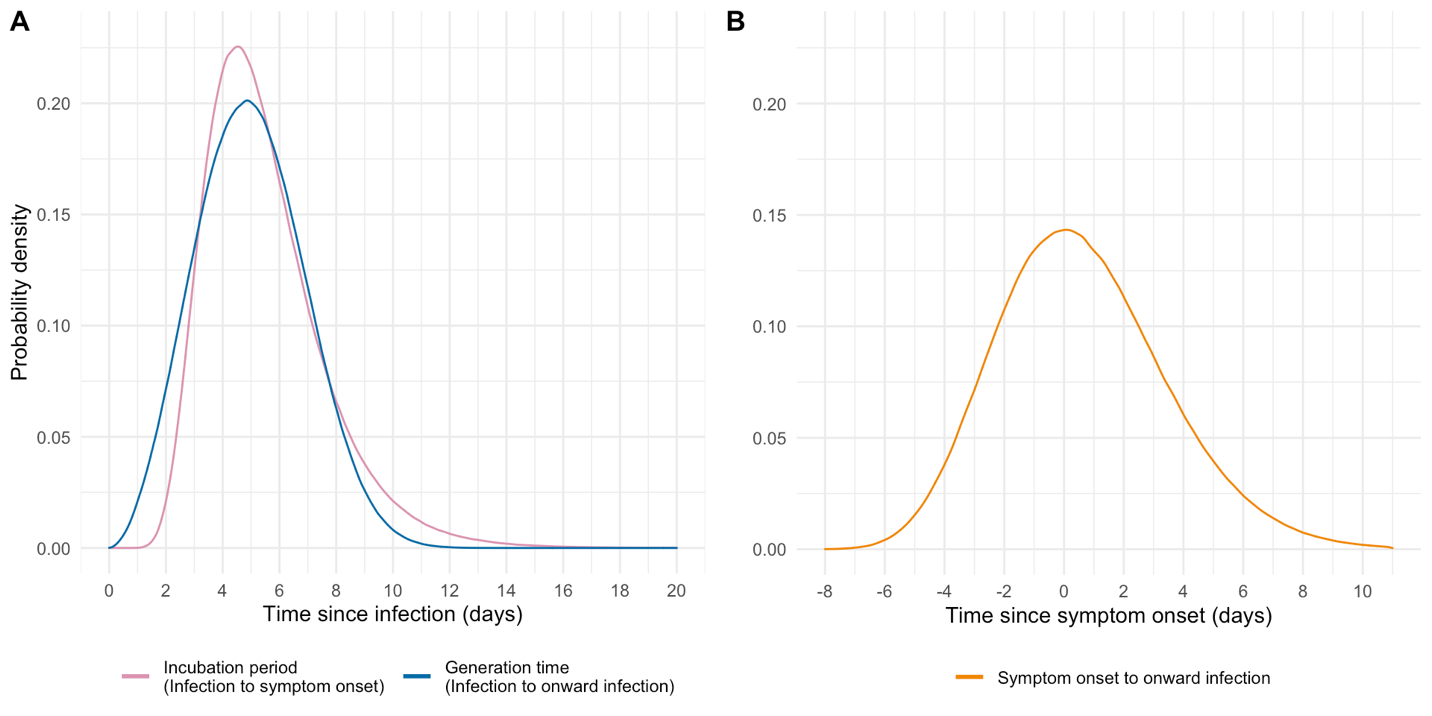


References:

1. He X, Lau EHY, Wu P, Deng X, Wang J, Hao X, et al. Temporal dynamics in viral shedding and transmissibility of COVID-19. Nat Med. 2020;26: 672–675. doi:10.1038/s41591-020-0869-5

2. Ferretti L, Wymant C, Kendall M, Zhao L, Nurtay A, Abeler-Dörner L, et al. Quantifying SARS-CoV-2 transmission suggests epidemic control with digital contact tracing. Science (80- ). 2020;368. doi:10.1126/science.abb6936

3. Lauer SA, Grantz KH, Bi Q, Jones FK, Zheng Q, Meredith HR, et al. The incubation period of coronavirus disease 2019 (CoVID-19) from publicly reported confirmed cases: Estimation and application. Ann Intern Med. 2020;172: 577–582. doi:10.7326/M20-0504
