# Supplementary material for: Transmission of community- and hospital-acquired SARS-CoV-2 in hospital settings in the UK: A cohort study
Source: PLoS Med. 2021 Oct 12;18(10):e1003816. doi: 10.1371/journal.pmed.1003816 (PMC8509983; doi:10.1371/journal.pmed.1003816)

Supplementary material S3 Figure

**Infection pressure from the community**

Infection pressure from the community was extrapolated from the number of patients with community-acquired SARS-CoV-2 infection admitted to the hospitals. This was used instead of detected number of COVID-19 cases reported in the community as the latter is distorted by substantial temporal variation in the community testing rate. The figure below shows the daily numbers of infected individuals by their symptom onset dates (grey bars). The blue line shows the smoothed numbers per day derived using a generalised additive model. We used the smoothed numbers as an independent variable in the models for patient and healthcare worker transmissions.


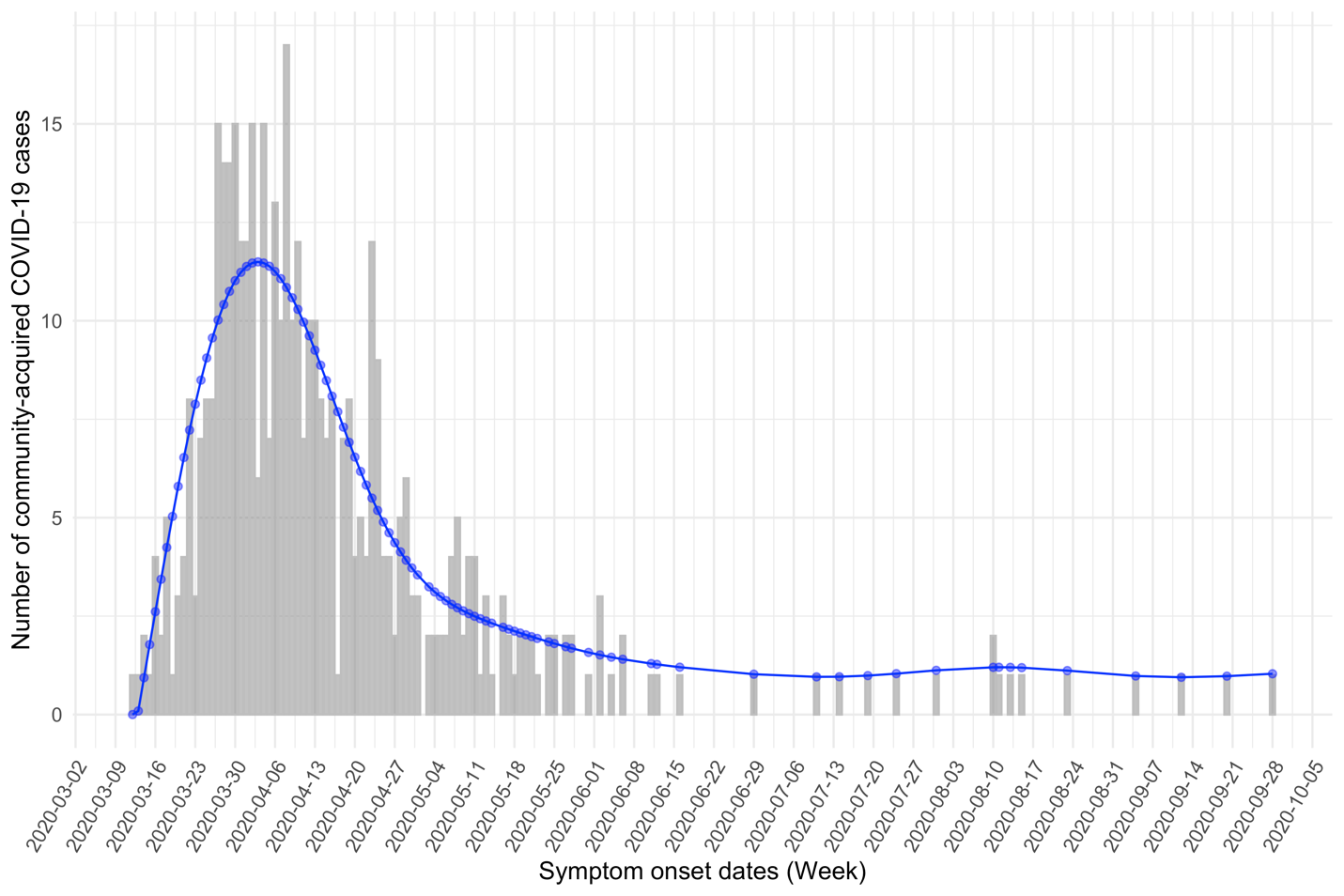

Supplement: S3 Fig — COVID-19, Coronavirus Disease 2019; SARS-CoV-2, Severe Acute Respiratory Syndrome Coronavirus 2. (DOCX) [file pmed.1003816.s004.docx]
