# Supplementary material for: Transmission of community- and hospital-acquired SARS-CoV-2 in hospital settings in the UK: A cohort study
Source: PLoS Med. 2021 Oct 12;18(10):e1003816. doi: 10.1371/journal.pmed.1003816 (PMC8509983; doi:10.1371/journal.pmed.1003816)

# Supplementary material S1 Text

## Logistic regression (Model 1) results

## Univariable analysis (Model 1*_univariable_*)

Table A: Univariable logistic regression results where the outcome is patient SARS-CoV-2 infection during the hospital stay (model *P*1*_univariable_*).

|  |  | |  | |  | | |  | | Incubation period | | | | |  | |  | | |  |
| --- | --- | --- | --- | --- | --- | --- | --- | --- | --- | --- | --- | --- | --- | --- | --- | --- | --- | --- | --- | --- |
|  |  | | **5 days** | | | | | | | **3 days** | | | | |  | | **7 days** | | |  |
| Characteristics | | **N** | | **OR^1^** | | **95% CI^1^** | **p-value^1^** | | **N** | | **OR^1^** | **95% CI^1^** | **p-value^1^** | **N** | | **OR^1^** | | **95% CI^1^** | | **p-value^1^** |
| Age | | 148,998 | | 1.04 | | 1.03, 1.05 | *<*0.001 | | 192,293 | | 1.03 | 1.02, 1.04 | *<*0.001 | 121,299 | | 1.04 | | 1.03, 1.06 | | *<*0.001 |
| Sex | | 148,998 | |  | |  |  | | 192,293 | |  |  |  | 121,299 | |  | |  | |  |
| Female | |  | | 1 | | — |  | |  | | 1 | — |  |  | | 1 | | — | |  |
| Male | |  | | 1.04 | | 0.75, 1.44 | 0.8 | |  | | 0.97 | 0.69, 1.35 | 0.8 |  | | 0.88 | | 0.62, 1.25 | | 0.5 |
| Ethnic group | | 119,511 | |  | |  |  | | 154,446 | |  |  |  | 97,130 | |  | |  | |  |
| White | |  | | 1 | | — |  | |  | | 1 | — |  |  | | 1 | | — | |  |
| Non-white | |  | | 0.17 | | 0.03, 0.53 | 0.013 | |  | | 0.00 | 0.00, 0.21 | 0.93 |  | | 0.18 | | 0.03, 0.57 | | 0.017 |
| Infectious patients with community-acquired SARS-CoV-2 on the same ward | | 150,884 | | 1.46 | | 1.34, 1.57 | *<*0.001 | | 194,205 | | 1.36 | 1.25, 1.47 | *<*0.001 | 123,159 | | 1.55 | | 1.38, 1.69 | | *<*0.001 |
| Infectious patients with hospital-acquired SARS-CoV-2 on the same ward | | 150,884 | | 2.77 | | 2.52, 3.02 | *<*0.001 | | 194,205 | | 3.13 | 2.83, 3.44 | *<*0.001 | 123,159 | | 2.60 | | 2.35, 2.87 | | *<*0.001 |
| Infectious healthcare worker on the same ward | | 142,644 | | 2.27 | | 2.06, 2.48 | *<*0.001 | | 182,772 | | 3.03 | 2.64, 3.44 | *<*0.001 | 116,750 | | 1.98 | | 1.83, 2.13 | | *<*0.001 |
| Hospital | | 150,884 | |  | |  |  | | 194,205 | |  |  |  | 123,159 | |  | |  | |  |
| Hospital A | |  | | 1 | | — |  | |  | | 1 | — |  |  | | 1 | | — | |  |
| Hospital B | |  | | 4.41 | | 2.72, 9.92 | *<*0.001 | |  | | 4.51 | 2.12, 10.70 | *<*0.001 |  | | 5.82 | | 2.71, 14.40 | | *<*0.001 |
| Hospital C | |  | | 2.10 | | 1.17, 4.71 | 0.024 | |  | | 2.53 | 1.31, 5.66 | 0.012 |  | | 2.38 | | 1.18, 5.69 | | 0.028 |
| Hospital D | |  | | 4.23 | | 2.00, 9.74 | *<*0.001 | |  | | 4.76 | 2.15, 11.5 | *<*0.001 |  | | 4.14 | | 1.78, 10.70 | | *<*0.002 |
| Type of ward | | 150,884 | |  | |  |  | | 194,205 | |  |  |  | 123,1595 | |  | |  | |  |
| General Ward | |  | | 1 | | — |  | |  | | 1 | — |  |  | | 1 | | — | |  |
| ICU/ HDU^2^ | |  | | 0.49 | | 0.19, 1.01 | 0.087 | |  | | 0.37 | 0.11, 0.87 | 0.05 |  | | 0.17 | | 0.03, 0.54 | | 0.014 |
| Phase | | 150,884 | |  | |  |  | | 194,205 | |  |  |  | 123,159 | |  | |  | |  |
| 1 | |  | | 1 | | — |  | |  | | 1 | — |  |  | | 1 | | — | |  |
| 2 | |  | | 3.00 | | 2.01, 4.40 | *<*0.001 | |  | | 4.24 | 2.85, 6.27 | *<*0.001 |  | | 2.50 | | 1.62, 3.76 | | *<*0.001 |
| 3 | |  | | 0.24 | | 0.16, 0.35 | *<*0.001 | |  | | 0.32 | 0.21, 0.48 | *<*0.001 |  | | 0.18 | | 0.11, 0.28 | | *<*0.001 |
| Calendar day | | 150,884 | | 1.02 | | 1.00, 1.04 | 0.017 | | 194,205 | | 1.02 | 1.00, 1.04 | 0.022 | 123,159 | | 1.00 | | 0.98, 1.02 | | 0.8 |
| Day of stay | | 150,884 | | 0.99 | | 0.98, 1.00 | 0.008 | | 194,205 | | 0.99 | 0.98, 1.00 | 0.039 | 123,159 | | 0.98 | | 0.97, 0.99 | | 0.001 |
| ^1^ OR = Odds Ratio, CI = Confidence Interval, p-values were calculated with the Wald test  ^2^ ICU/ HDU = Intensive care units/ High dependency units | | | | | | | | | | | | | | |  | |  | |  |  |

Table B: Univariable logistic regression results where the outcome is healthcare worker COVID-19 infection during the hospital stay (model *H*1*_univariate_*).

|  | Incubation period | | | | | | | | | | | | | |
| --- | --- | --- | --- | --- | --- | --- | --- | --- | --- | --- | --- | --- | --- | --- |
|  | **5 days** | | | | | **3 days** | | | | | **7 days** | | | |
| Characteristics | **N** | **OR^1^** | **95% CI^1^** | **p-value^1^** | **N** | | **OR^1^** | **95% CI^1^** | **p-value^1^** | **N** | | **OR^1^** | **95% CI^1^** | **p-value^1^** |
| Age | 1,350,592 | 1.00 | 0.99, 1.01 | 0.9 | 1,361,784 | | 1.00 | 0.99, 1.01 | 0.9 | 1,339,400 | | 1.00 | 0.99, 1.01 | 0.9 |
| Sex | 1,350,592 |  |  |  | 1,361,784 | |  |  |  | 1,339,400 | |  |  |  |
| Female |  | 1 | — |  |  | | 1 | — |  |  | | 1 | — |  |
| Male |  | 0.97 | 0.80, 1.18 | 0.8 |  | | 0.97 | 0.80, 1.18 | 0.8 |  | | 0.97 | 0.80, 1.18 | 0.8 |
| Role | 1,350,592 |  |  |  | 1,361,784 | |  |  |  | 1,339,400 | |  |  |  |
| Doctor |  | 1 | — |  |  | | 1 | — |  |  | | 1 | — |  |
| Nurse |  | 1.47 | 1.18, 1.86 |  | *<*0.001 | | 1.47 | 1.18, 1.86 | *<*0.001 |  | | 1.47 | 1.18, 1.86 | *<*0.001 |
| Allied Health |  | 1.03 | 0.80, 1.34 | 0.8 |  | | 1.03 | 0.80, 1.34 | 0.8 |  | | 1.03 | 0.80, 1.34 | 0.8 |
| Non-clinical staff |  | 0.95 | 0.70, 1.28 | 0.7 |  | | 0.95 | 0.70, 1.28 | 0.7 |  | | 0.95 | 0.70, 1.28 | 0.7 |
| Infectious cases in the community | 1,350,592 | 1.28 | 1.26, 1.30 | *<*0.001 | 1,361,784 | | 1.29 | 1.27, 1.31 | *<*0.001 | 1,339,400 | | 1.26 | 1.24, 1.29 | *<*0.001 |
| Infectious patients with community-acquired SARS-CoV-2 on the same ward | 1,063,844 | 1.33 | 1.26, 1.39 | *<*0.001 | 1,074,079 | | 1.25 | 1.21, 1.28 | *<*0.001 | 1,055,216 | | 1.38 | 1.27, 1.49 | *<*0.001 |
| Infectious patients with hospital-acquired SARS-CoV-2 on the same ward | 1,063,844 | 2.23 | 2.08, 2.36 | *<*0.001 | 1,074,079 | | 2.40 | 2.25, 2.55 | *<*0.001 | 1,055,216 | | 2.17 | 2.04, 2.30 | *<*0.001 |
| Infectious healthcare worker on the same ward | 1,350,592 | 1.96 | 1.87, 2.05 | *<*0.001 | 1,361,784 | | 2.36 | 2.21, 2.52 | *<*0.001 | 1,374,267 | | 1.77 | 1.71, 1.84 | *<*0.001 |
| Hospital | 1,350,592 |  |  |  | 1,361,784 | |  |  |  | 1,339,400 | |  |  |  |
| Hospital A |  | — | — |  |  | | — | — |  |  | | — | — |  |
| Hospital B |  | 1.95 | 1.46, 2.59 | *<*0.001 |  | | 1.95 | 1.46, 2.59 | *<*0.001 |  | | 1.95 | 1.46, 2.59 | *<*0.001 |
| Hospital C |  | 1.15 | 0.93, 1.45 | 0.2 |  | | 1.15 | 0.93, 1.45 | 0.2 |  | | 1.15 | 0.93, 1.45 | 0.2 |
| Hospital D |  | 1.68 | 1.18, 2.37 | 0.003 |  | | 1.68 | 1.18, 2.37 | 0.003 |  | | 1.68 | 1.18, 2.37 | 0.003 |
| Type of ward | 1,350,592 |  |  |  | 1,361,784 | |  |  |  | 1,339,400 | |  |  |  |
| General Ward |  | 1 | — |  |  | | 1 | — |  |  | | 1 | — |  |
| ICU/ HDU^2^ |  | 0.61 | 0.44, 0.81 | 0.001 |  | | 0.61 | 0.44, 0.81 | 0.001 |  | | 0.61 | 0.44, 0.81 | 0.001 |
| Phase | 1,350,592 |  |  |  | 1,361,784 | |  |  |  | 1,339,400 | |  |  |  |
| 1 |  | 1 | — |  |  | | 1 | — |  |  | | 1 | — |  |
| 2 |  | 2.59 | 2.14, 3.13 | *<*0.001 |  | | 1.91 | 1.57, 2.32 | *<*0.001 |  | | 1.91 | 1.57, 2.32 | *<*0.001 |
| 3 |  | 0.40 | 0.33, 0.48 | *<*0.001 |  | | 0.33 | 0.27, 0.39 | *<*0.001 |  | | 0.34 | 0.28, 0.41 | *<*0.001 |
| Calendar day | 1,350,592 | 0.99 | 0.98, 1.00 | 0.012 | 1,361,784 | | 0.98 | 0.97, 0.99 | *<*0.001 | 1,339,400 | | 1.00 | 0.99, 1.01 | 0.8 |
| ^1^ OR = Odds Ratio, CI = Confidence Interval, p-values were calculated with the Wald test  ^2^ ICU/ HDU = Intensive care units/ High dependency units | | | | | | | | | | | | | | |

## Generalised additive model (Model 2) results

**Daily risk of patient nosocomial SARS-CoV-2 infection (model** *P*2**)**

Figure A. Daily probability of having a first positive SARS-CoV-2 PCR test during hospitalisation. The coloured lines represent the daily probabilities of having the first positive SARS-CoV-2 PCR test throughout a patient’s hospitalisation for months from February to September 2020. These probabilities are predictions from the generalised additive model with a logit link, with the binary outcome of assumed acquisition (yes/no) on each day as the dependent variable, and infectious patients and healthcare workers as the independent variables. Infectious patients were classified as having nosocomial SARS-CoV-2 infections with the assumption of a 5-day incubation period.


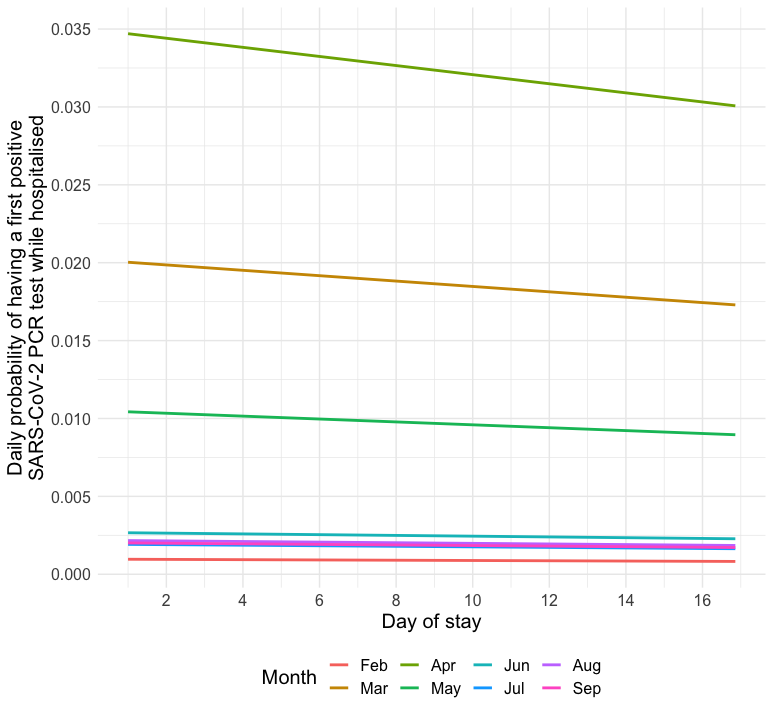


## Generalised linear model with identity link (Model 3)

### Model comparison

To quantify the daily transmission risk posed by infectious patients and healthcare workers, we used a generalised linear mixed model with an identity link, thus allowing for the daily probability of infection to scale linearly with infection pressure from healthcare workers and patients and for their effects to be additive. Two models, one with interaction terms between the phases and forces of infection from patients and healthcare workers, and one without the interaction terms, were compared. Between these transmission models, the model with the best fit to data by WAIC was the one without interaction terms, which has an intercept (*α*), representing the infection risk not explained by covariates, and slopes (*beta*) which represent the infection risk associated with infectious patients (community- and hospital-acquired) and healthcare workers. We allowed both the intercepts (*α)* and slopes (*beta*) to vary by ward in order to account for the differences in the ward set-ups. The final estimates presented are the mean and 95% credible intervals of estimates obtained for each ward.

Table C: Comparison of widely applicable information criterion between a model with no interaction terms between phases and infection pressure from patients and healthcare workers versus a model with interaction terms.

| Susceptible host | Transmission model | Parameters | Priors | WAIC † |
| --- | --- | --- | --- | --- |
| Patients | No interactions terms between phases and infection pressure from patients and healthcare  workers | *α* (intercept)  *β*_1_ (probability of transmission from infectious patients with community-acquired SARS-CoV-2)  *β*_2_ (probability of transmission from infectious patients with hospital-acquired SARS-CoV-2)  *β*_3_ (probability of transmission from infected healthcare workers) | *Half normal*(0*,7)* | -284,770 |
|  | Interactions terms between phases and infection pressure from patients and healthcare workers | *α* (intercept)  *β*_1_ (probability of transmission from infectious patients with community-acquired SARS-CoV-2)  *β*_2_ (probability of transmission from infectious patients with hospital-acquired SARS-CoV-2)  *β*_3_ (probability of transmission from infected healthcare workers)  *γ*_1_ (probability of transmission in phase 2)  *γ*_2_ (probability of transmission in phase 3) | *Half normal*(0*,7)* | -142,632 |
| Healthcare workers | No interactions terms between phases and infection pressure from patients and healthcare  workers | *α* (intercept)  *β*_1_ (probability of transmission from infectious patients with community-acquired SARS-CoV-2)  *β*_2_ (probability of transmission from infectious patients with hospital-acquired SARS-CoV-2)  *β*_3_ (probability of transmission from infected healthcare workers)  *β_4_* (probability of transmission from community cases) | *Half normal*(0*,7)* | -19,285 |
|  | Interactions terms between phases and infection pressure from patients and healthcare workers | *α* (intercept)  *β*_1_ (probability of transmission from infectious patients with community-acquired SARS-CoV-2)  *β*_2_ (probability of transmission from infectious patients with hospital-acquired SARS-CoV-2)  *β*_3_ (probability of transmission from infected healthcare workers)  *β_4_* (probability of transmission from community cases)  *γ*_1_ (probability of transmission in phase 2)  *γ*_2_ (probability of transmission in phase 3) | *Half normal*(0*,7)* | -10,484 |

† Widely applicable information criterion (WAIC).

### Model assessment of the main analysis models

Prior distributions were selected to be weakly informative half-normal distributions, such that the prior values are kept positive. We assessed the models using measures of Markov chain convergence including effective sample sizes and *R*ˆ which indicate if the chains had run for long enough and had mixed well.

Plots of iterations vs. sampled values for model parameters in the MCMC chains. The three different chains are plotted using different colours.

In the main analysis model where the outcome is hospital-acquired SARS-CoV2 infection amongst the patients, the *R*ˆ values were about 1 and the minimum effective sample size was 1700 across all parameters. The chains’ mixing is shown below.

Figure B. Model where outcome is patient SARS-CoV-2 infection acquired during hospitalisation. First plot of each parameter, representing a single ward is shown.


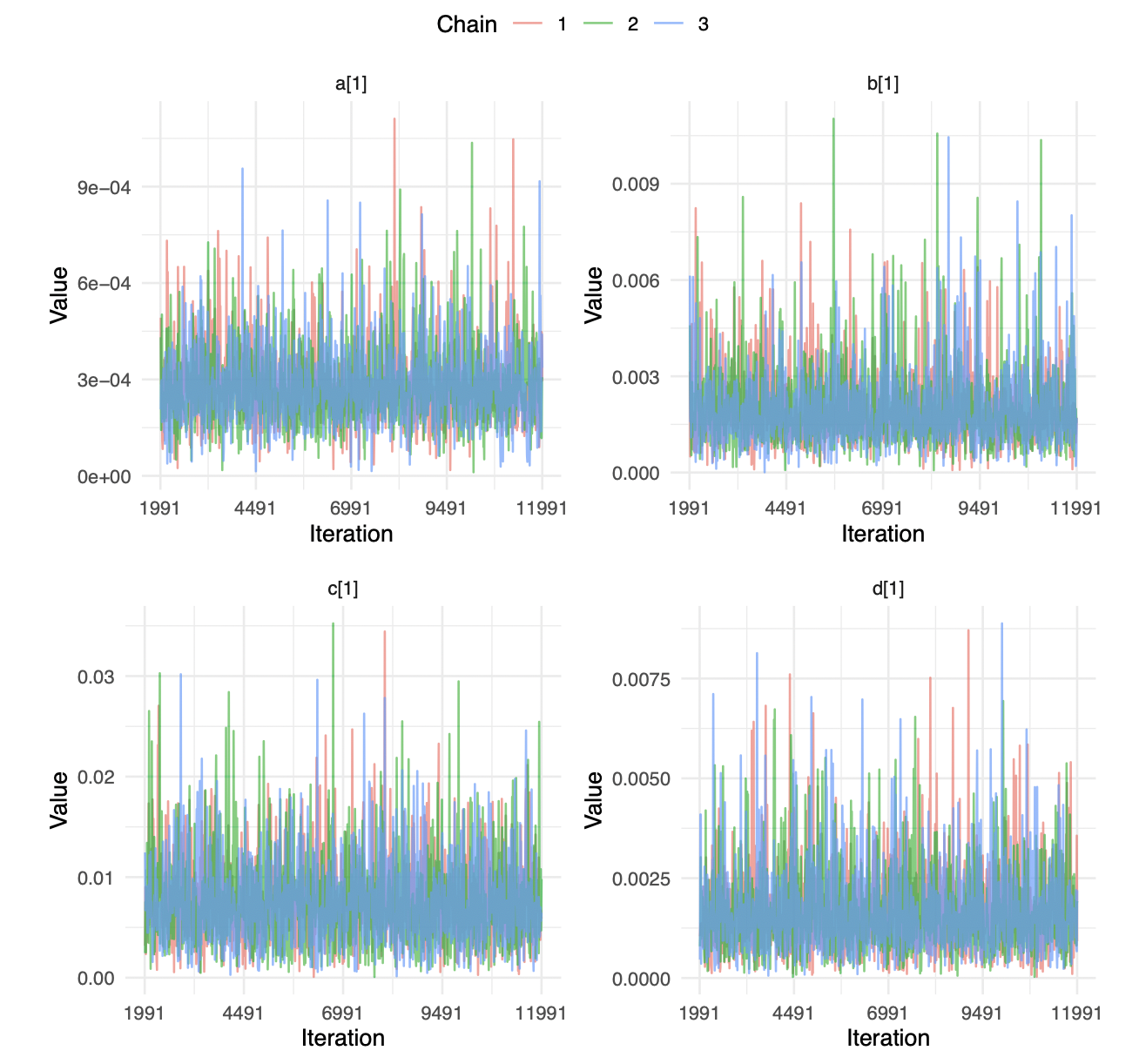


In the main analysis model where the outcome is hospital-acquired SARS-CoV2 infection amongst the HCW, the *R*ˆ values were about 1 and the minimum effective sample size was 1500 across all parameters. The chains’ mixing is shown below.

Figure C: Model where outcome is healthcare worker SARS-CoV-2 infection. First plot of each parameter, representing a single ward is shown.


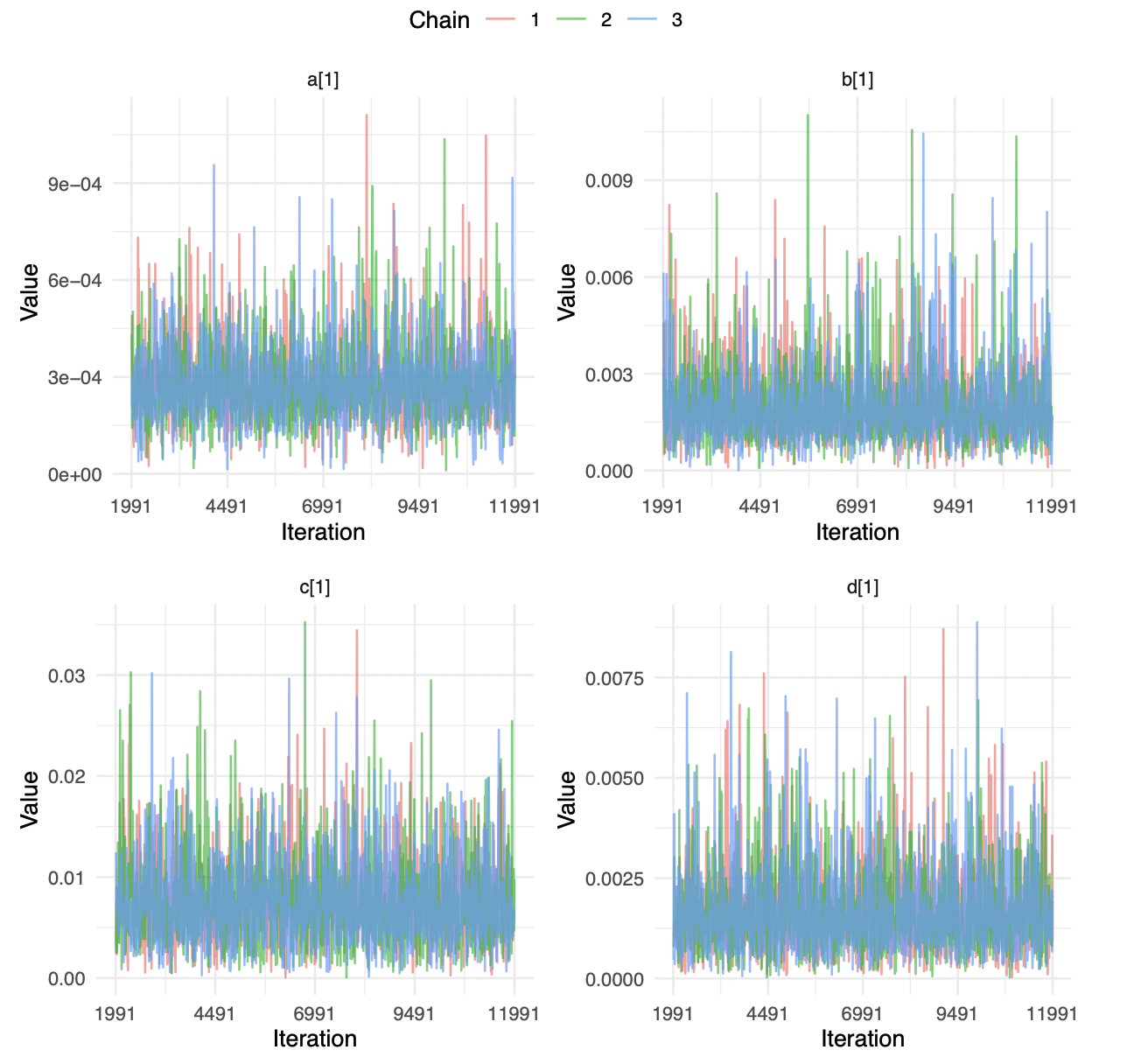

Supplement: S1 Text — (DOCX) [file pmed.1003816.s005.docx]
