# Supplementary material for: Transmission of community- and hospital-acquired SARS-CoV-2 in hospital settings in the UK: A cohort study
Source: PLoS Med. 2021 Oct 12;18(10):e1003816. doi: 10.1371/journal.pmed.1003816 (PMC8509983; doi:10.1371/journal.pmed.1003816)
Supplement: S2 Text — (DOCX) [file pmed.1003816.s006.docx]

# Supplementary material S2 Text

## Generalised linear model with identity link (Model 3) model Sensitivity analysis

### Sensitivity to infectiousness scaled by day of infection according to incubation period

Table A: The main analysis considers infectiousness to be binary, i.e., absolute numbers of infectious patients and healthcare workers in a ward on a particular day were used. Sensitivity analysis considered infectiousness to be scaled according to the time since the day of infection which, in turn, is based on an assumed incubation period of five days. This scaling of the number of infectious patients and healthcare workers in a ward on a particular day makes use of the relative infectiousness distribution derived by He *et al* (S6 Fig) such that the sum of daily terms for a single infected patient who was present in the ward throughout their entire infectious period would equal one. Hence, the scaled parameters are an order of magnitude higher than the binary infectiousness model estimates.

| **Infectious population posing transmission risk** | **Additional risk of acquiring nosocomial SARS-CoV-2 (%)**  **(mean, 95% credible interval)** | | | |
| --- | --- | --- | --- | --- |
|  | **For susceptible patients** | | **For susceptible healthcare workers** | |
|  | **Binary infectiousness**  **(main analysis)** | **Scaled infectiousness** | **Binary infectiousness**  **(main analysis)** | **Scaled infectiousness** |
| **Background infection risk including undetected cases** | 0.03 (0.02-0.03) | 0.03 (0.03-0.04) | 0.01 (0.01-0.01) | 0.01 (0.01-0.01) |
| **Infectious cases in the community** | -- | -- | 0.01 (0.01-0.02) | 0.01 (0.01-0.02) |
| **Patients who acquired the infection from the community on the same ward** | 0.20 (0.16-0.22) | 1.96 (1.6-2.23) | 0.02 (0.02-0.02) | 0.24 (0.2-0.28) |
| **Patients who acquired the infection from the hospital on the same ward** | 0.75 (0.55-0.95) | 6.56 (5.33-7.94) | 0.08 (0.06-0.1) | 0.72 (0.56-0.9) |
| **Healthcare workers on the same ward** | 0.17 (0.13-0.22) | 1.45 (1.01-1.91) | 0.08 (0.03-0.16) | 0.61 (0.26-1.21) |

### Sensitivity to choice of prior distributions

Table B: Prior distributions for the various parameters were changed to test the sensitivity of the estimates to the choice of prior distributions.

| **Infectious population posing transmission risk** | **Additional risk of acquiring nosocomial COVID-19 (%)**  **(mean, 95% credible interval)** | | | |
| --- | --- | --- | --- | --- |
|  | **For susceptible patients** | | **For susceptible healthcare workers** | |
|  | *α: half normal*(0*, 7)*  *β: half normal*(0*, 7*)  **(main analysis)** | *α: half normal*(0*, 7)*  *β: half normal*(0.01*, 3*) | *α: half normal*(0*, 7)*  *β: half normal*(0*, 7*)  **(main analysis)** | *α: half normal*(0*, 7)*  *β: half normal*(0.01*, 3*) |
| **Background infection risk including undetected cases** | 0.03 (0.02-0.03) | 0.03 (0.02-0.03) | 0.01 (0.01-0.01) | 0.01 (0.01-0.01) |
| **Infectious cases in the community** | -- | -- | 0.01 (0.01-0.02) | 0.01 (0.01-0.02) |
| **Patients who acquired the infection from the community on the same ward** | 0.20 (0.16-0.22) | 0.20 (0.16-0.22) | 0.02 (0.02-0.02) | 0.02 (0.02-0.02) |
| **Patients who acquired the infection from the hospital on the same ward** | 0.75 (0.55-0.95) | 0.74 (0.56-0.95) | 0.08 (0.06-0.1) | 0.08 (0.06-0.1) |
| **Healthcare workers on the same ward** | 0.17 (0.13-0.22) | 0.16 (0.13-0.22) | 0.08 (0.03-0.16) | 0.08 (0.03-0.16) |
