# Supplementary material for: Transmission of community- and hospital-acquired SARS-CoV-2 in hospital settings in the UK: A cohort study
Source: PLoS Med. 2021 Oct 12;18(10):e1003816. doi: 10.1371/journal.pmed.1003816 (PMC8509983; doi:10.1371/journal.pmed.1003816)
Supplement: S3 Text — (DOCX) [file pmed.1003816.s007.docx]

# Supplementary material S3 Text

## Model codes

Code block A: Logistic regression model using patient or healthcare worker infection as the dependent variable.

glm.logistic.model_patient <- glm(outcome ~ age + sex + ‘ethnic group’ + ‘infectious patients on the same ward (community-acquired)‘ + ‘infectious patients on the same ward (hospital-acquired)’ + ‘infectious staff on the same ward’ + ‘hospital id’ + ‘type of ward’ + phase + splines::ns(‘Calendar day‘, 2), data = data, family = ’binomial’)

glm.logistic.model_hcw <- glm(outcome ~ age + sex + ‘ethnic group’ + ‘infectious cases in the community’ + ‘infectious patients on the same ward (community-acquired)‘ + ‘infectious patients on the same ward (hospital-acquired)’ + ‘infectious staff on the same ward’ + ‘hospital id’ + ‘type of ward’ + phase + splines::ns(‘Calendar day‘, 2), data = data, family = ’binomial’)

Code block B: Generalised additive model using patient infection as the dependent variable, and taking demographic factors, calendar day, day of hospitalisation, and number of infectious patients and healthcare workers on the same ward per day as independent variables.

gam.model <- gam(outcome ~ s(‘calendar day’) + s(‘day of stay’) + s(‘patient absolute nosocomial’) + s(‘HCW absolute’) + age + sex + ‘ethnic group’ + ‘type of ward‘ + ‘hospital id’ + phase, family=binomial(link = ’logit’), data = data)

Code block C: Generalised linear mixed model with identity link using patient infection as the dependent variable (implemented in R with JAGS using a non-centred parameterisation).

glm.identity.model <- function(){

# Likelihood:

for (i in 1:N){

outcome[i] ~ dbern(mu[i])

mu[i] <- a[admission_ward_index[i]] + b[admission_ward_index[i]] * ‘Infectious patients on the same ward (community-acquired)‘[i] + c[admission_ward_ index[i]] * ‘Infectious patients on the same ward ( hospital-acquired)‘[i] + d[admission_ward_index[i]] * ‘ Infectious staff on the same ward‘[i]

# For WAIC computation

loglike[i] <- dbin(outcome[i], mu[i], 1)

}

for (w in 1:N_ward){

a[w] <- a0 + aprimed[w] * sigma.a

b[w] <- b0 + bprimed[w] * sigma.b

c[w] <- c0 + cprimed[w] * sigma.c

d[w] <- d0 + dprimed[w] * sigma.d

aprimed[w] ~ dnorm(0, 1);T(0,);

bprimed[w] ~ dnorm(0, 1);T(0,);

cprimed[w] ~ dnorm(0, 1);T(0,);

dprimed[w] ~ dnorm(0, 1);T(0,);

}

# Priors:

a0 ~ dnorm(0, 0.1);T(0,); sigma.a ~ dunif(0, 05);

b0 ~ dnorm(0.01, 0.1);T(0,); sigma.b ~ dunif(0, 1);

c0 ~ dnorm(0.01, 0.05);T(0,); sigma.c ~ dunif(0, 1);

d0 ~ dnorm(0.01, 0.05);T(0,); sigma.d ~ dunif(0, 1);

}
