# Supplementary material for: Transmission of community- and hospital-acquired SARS-CoV-2 in hospital settings in the UK: A cohort study
Source: PLoS Med. 2021 Oct 12;18(10):e1003816. doi: 10.1371/journal.pmed.1003816 (PMC8509983; doi:10.1371/journal.pmed.1003816)
Supplement: S4 Text — (DOCX) [file pmed.1003816.s008.docx]

# Supplementary material S4 Text

## Details of real-time polymerase chain reaction (RT-PCR) performed with combined nasal and oropharyngeal swabs

RT-PCR was performed using the Public Health England SARS-CoV-2 assay (targeting the RdRp gene), one of five commercial assays: Abbott RealTime (targeting RdRp and N genes; Abbott, Maidenhead, UK), Altona RealStar (targeting E and S genes; Altona Diagnostics, Liverpool, UK), Cepheid Xpert® Xpress SARS-CoV-2 (targeting N2 and E; Cepheid, California, USA), BioFire® Respiratory 2.1 (RP2.1) panel with SARS-CoV-2 (targeting ORF1ab and ORF8; Biofire diagnostics, Utah, USA), Thermo Fisher TaqPath assay (targeting S and N genes, and ORF1ab; Thermo Fisher, Abingdon, UK) or using the ABI 7500 platform (Thermo Fisher, Abingdon, UK) with the US Centers for Disease Control and Prevention Diagnostic Panel of two probes targeting the N gene.
