# Supplementary material for: Transmission of community- and hospital-acquired SARS-CoV-2 in hospital settings in the UK: A cohort study
Source: PLoS Med. 2021 Oct 12;18(10):e1003816. doi: 10.1371/journal.pmed.1003816 (PMC8509983; doi:10.1371/journal.pmed.1003816)
Supplement: S5 Text — (DOCX) [file pmed.1003816.s009.docx]

**Supplementary material S5 Text**

**Oxford COVID infection review team**

University of Oxford Medical School

- Hannah Chase
- Ishta Sharma
- Sarah Peters
- Archie Lodge
- Sai Arathi Parepalli
- Raghav Sudarshan
- Hannah Callaghan
- Imogen Vorley

Oxford University Hospitals

- Omar Risk
- Tamsin Cargill
- Maria Tsakok
- Grace Barnes
- Josh Hamblin
- Jenny Tempest-Mitchell
- Ashley Elder
- Danica Fernandes
- Bara'a Elhag
- Edward David
- Rumbi Mutenga
- Dylan Riley
- Gurleen Kaur
- Emel Yildirim
- Naomi Hudson
