# Supplementary material for: Transmission of community- and hospital-acquired SARS-CoV-2 in hospital settings in the UK: A cohort study
Source: PLoS Med. 2021 Oct 12;18(10):e1003816. doi: 10.1371/journal.pmed.1003816 (PMC8509983; doi:10.1371/journal.pmed.1003816)
Supplement: Attachment — Submitted filename: mo.pdf [file pmed.1003816.s010.pdf]

This paper uses routine data to examine infection risk in hospitals in Oxfordshire.

I was asked for a statistical report and I interpret that to include all aspects of the design and conduct of the study.

Points of detail

Page 7 It is not clear to me what the force of '... who were on the same wards ... ' is. Does this mean that they had to be on the same ward for their whole stay? But that seems to contradict the inclusion of location as time–varying later on this page. Table 2 seems to suggest that staff were only included if they served only one ward. If this is true is that not a substantial limitation as many categories of staff service multiple locations and even multiple hospitals?

Page 9 The definitions of community–acquired and hospital–acquired do not seem to be together exhaustive. If this is true what happened to the rest and is that a limitation?

Page 11 I think it would be usual to specify the risk factors which were going to be included in the models here. I know we can find out in Table 3 but that raises the question of whether all of the ones reported there were pre–specified or selected by some data–driven procedure. I assume there was in fact no selection based on Tables S1 and S2 but it would be good to have it confirmed explicitly.

Page 12 I am afraid I do not understand what relevance a re–admission has. If, as page 8 informs us, they can only be infected once then how does re–admission come into the picture?

Page 13 Collapsing together all other ethnic groups lacks a clear justification. If we take examples of heritages represented in the UK like Nigeria, Bangladesh, or Hong Kong, is there any reason to suppose they are all alike and different from white people? Table 3 even collapses Other into non–White. I know the media and official statistics regularly collapse all non–White but that does not make it make scientific sense.

Page 17, Table 3 This might be better in landscape so the confidence intervals did not span rows. It is already broken over two pages so that is not an issue. The same applies to Table 4.

Page 18 I am unconvinced that being multiplicative is a disadvantage of

logistic models, that is just what they do, it is a feature.

References I know this is very picky but some of these have names which lack their appropriate initial capitals.

Figure 3 This suffers from over–printing in the upper right panel. It might be worth experimenting with eliminating the fill colour.

Points of more substance

Additive versus multiplicative

The abstract concentrates on the additive model but in the results it is presented as an alternative rather than as the primary model. Absolute risk as opposed to relative has many advantages, I am not objecting to the choice in the abstract, but it seems inconsistent to make the multiplicative the main focus in the results.

What are UK hospitals like?

If this is going to be useful to readers in other countries do they need to know more about the usual layout of hospital wards in the UK or in these hospitals specifically? I fortunately have limited knowledge of this but my impression is that four– and six–bedded bays are common here so results might be different in countries which have single rooms or Nightingale wards.

Summary

Mostly points for clarification.

Michael Dewey
