# Supplementary material for: Transmission of community- and hospital-acquired SARS-CoV-2 in hospital settings in the UK: A cohort study
Source: PLoS Med. 2021 Oct 12;18(10):e1003816. doi: 10.1371/journal.pmed.1003816 (PMC8509983; doi:10.1371/journal.pmed.1003816)
Supplement: Attachment — Submitted filename: covidtransmission_plosreviewreply.docx [file pmed.1003816.s011.docx]

Professor Raffaella Bosurgi

*Editor-in-Chief*

*PLoS Medicine*

30 July 2021

Dear Professor Bosurgi,

**Re: Manuscript submission: “Identifying the drivers of SARS-CoV-2 transmission in the hospital setting: an observational cohort study” for PLoS Medicine**

Thank you very much for the reviewers’ and the editorial board’s insightful comments, and for giving us the opportunity to revise our manuscript. Please find the accompanying revised manuscript for your review and consideration. Our responses to the comments are as follows.

- **Comments from the Academic Editor**

*I think the study is interesting and relevant. What is maybe most problematic is the assumption that infected HCW were infected in the hospital. I believe that most transmission in that time period took place in the community, and I would guess that most HCWs got infected in the community. I agree with the reviewers that there needs to be more information on how the setup is of the hospitals from which the data came, and what the implemented infection prevention strategies were. Also in particular, how these hospitals dealt with COVID patients and with HCWs showing any symptoms.*

Thank you for these comments. In the revised manuscript, we have added infection pressure from the community cases in the models where healthcare workers were the susceptible population. This has been elaborated in the Methods section: “In the model where the susceptible population was HCW, we also included community cases as an additional infection pressure. To circumvent testing bias due to the large increase in community testing capacity during the study period, the numbers of community cases on each day were identified using the number of community-acquired SARS-CoV-2 infections admitted to the hospital in the following week.” All the respective results in the text, tables and graphs have been updated as well with results from models that now adjust for community infection pressure. This new analysis has not led to any substantive changes in our key findings.

We have added a paragraph in the Methods section, Infection prevention and control measures subsection: “The hospitals consist of a mix of side-rooms and multi-occupancy bays of two to four individuals. Ventilation in these wards was largely via natural ventilation with windows. Known SARS-CoV-2 infected patients were cohorted into standard rooms. There were no airborne isolation rooms. The hospitals’ infection prevention and control strategies were implemented following the UK national guidelines. Since the first cases of COVID-19, HCWs showing compatible symptoms were required to immediately isolate and obtain a PCR test. Patients were encouraged to wear masks at all times, especially if leaving their immediate bed area for an investigation. Visitors were generally not permitted during the pandemic period.”

- **Requests from the editors**

*General comments: Throughout the paper, please adapt reference call-outs to the following style: "... since symptom onset [21,26]." (noting the absence of spaces within the square brackets).*

We have adapted the reference call-outs as requested.

*Title: Please revise your title according to PLOS Medicine's style. Please place the study design in the subtitle (ie, after a colon). We suggest “Transmission of community- and hospital-acquired SARS-CoV-2 in the hospital setting: a cohort study” or similar*

We have changed the title as suggested.

*Abstract Background: Provide expand upon the context of why the study is important. The final sentence should clearly state the study question.*

We have expanded the abstract background to include the context and the study question as follows: “Nosocomial spread of SARS-CoV-2 has been widely reported, but the transmission pathways amongst patients and healthcare workers are unclear. Identifying the risk factors and drivers for these nosocomial transmissions are critical for infection prevention and control interventions.”

*Abstract Methods and Findings: Please specify the study design.*

We have added the study design to the abstract methods and findings as follows: “This is an observational cohort study using data from four teaching hospitals in Oxfordshire, UK, from January to October 2020.”

*Abstract Methods and Findings: Please provide brief demographic details of the study population (e.g. sex, age, ethnicity, etc)*

We have included a brief demographic details for both the patients and health workers in the abstract methods and findings as follows: “Nine-hundred and twenty of 66184 patients who were hospitalised during the study period had a positive SARS-CoV-2 PCR test within the same period (1%). The mean age was 68 years, 49% were females and 68% were from the white ethnic group.”, and, “Amongst the 5596 healthcare workers, 615 (11%) tested positive during the study period using PCR or serological tests. The mean age was 40 years, 79% were females and 50% were nurses.”

*Abstract Methods and Findings: Please define CrI at first use.*

We have defined CrI as first use in the abstract methods and findings.

*Abstract Methods and Findings: Should eight infections per 1000 susceptible patients also be per day (as for 2/1000 susceptible patients/day, mentioned later)?*

Yes, to improve clarity, we have included the same expression in the brackets as follows: “For susceptible patients, one day in the same ward with another patient with hospital-acquired SARS-CoV-2 was associated with an additional eight infections per 1000 susceptible patients (95% Credible interval (CrI) 6-10/1000 susceptible patients/day).”

*Abstract Methods and Findings: In the last sentence of the Abstract Methods and Findings section, please describe 2-3 of the main limitation(s) of the study's methodology.*

We have added the main limitations to the abstract methods and findings as follows: “The main limitations were that the symptom onset dates and healthcare worker absence days were not available. These assumptions were tested in sensitivity analysis which showed broadly similar results.”

*Abstract Conclusions: Please begin your Abstract Conclusions with "In this study, we observed ..." or similar, to summarize the main findings from your study, emphasizing what is new without overstating your conclusions.*

We have expanded the abstract conclusions as follows: “In this study, we observed that exposure to patients with hospital-acquired SARS-CoV-2 poses a substantial infection risk to both healthcare workers and other hospitalised patients.”

*Author Summary: At this stage, we ask that you include a short, non-technical Author Summary of your research to make findings accessible to a wide audience that includes both scientists and non-scientists. The Author Summary should immediately follow the Abstract in your revised manuscript. This text is subject to editorial change and should be distinct from the scientific abstract. Please see our author guidelines for more information:* [*https://imsva91-ctp.trendmicro.com:443/wis/clicktime/v1/query?url=https%3a%2f%2fjournals.plos.org%2fplosmedicine%2fs%2frevising%2dyour%2dmanuscript%23loc%2dauthor%2dsummary&umid=02BBB362-C652-6305-80BF-1A5D7D4A4AA4&auth=6e3fe59570831a389716849e93b5d483c90c3fe4-4fbd0cc5600d4e15c3b5da706b6cbc5bc62d6afb*](https://imsva91-ctp.trendmicro.com:443/wis/clicktime/v1/query?url=https%3a%2f%2fjournals.plos.org%2fplosmedicine%2fs%2frevising%2dyour%2dmanuscript%23loc%2dauthor%2dsummary&umid=02BBB362-C652-6305-80BF-1A5D7D4A4AA4&auth=6e3fe59570831a389716849e93b5d483c90c3fe4-4fbd0cc5600d4e15c3b5da706b6cbc5bc62d6afb) *In the final bullet point of ‘What Do These Findings Mean?’, please describe the main limitation(s) of the study in non-technical language.*

We have included an Author summary as requested in the revised manuscript.

*Methods: Did your study have a prospective protocol or analysis plan? Please state this (either way) early in the Methods section. If a prospective analysis plan (from your funding proposal, IRB or other ethics committee submission, study protocol, or other planning document written before analyzing the data) was used in designing the study, please include the relevant prospectively written document with your revised manuscript as a Supporting Information file to be published alongside your study, and cite it in the Methods section. A legend for this file should be included at the end of your manuscript. If no such document exists, please make sure that the Methods section transparently describes when analyses were planned, and if/when reported analyses differed from those that were planned. Changes in the analysis-- including those made in response to peer review comments-- should be identified as such in the Methods section of the paper, with rationale. If a reported analysis was performed based on an interesting but unanticipated pattern in the data, please be clear that the analysis was data-driven.*

Our study did not have a document outlining the prospective protocol or analysis plan. We have explained this in the Methods section, Statistical models subsection: “The analysis was data-driven.”

*Please provide the name(s) of the institutional review board(s) that provided ethical approval for your study.*

The study was approved by the Infections in Oxfordshire Research Database (IORD) which has generic Research Ethics Committee, Health Research Authority and Confidentiality Advisory Group approvals. This has been explained in the Methods section, Study cohort subsection.

*Thank you for providing a completed STROBE checklist in the Supporting Information. Please add the following statement, or similar, to the Methods: "This study is reported as per the Strengthening the Reporting of Observational Studies in Epidemiology (STROBE) guideline (S1 Checklist)." Please revise your checklist to use section and paragraph numbers, rather than page numbers.*

We have included the above suggested sentence to the methods section. The checklist has also been updated to use section and paragraph numbers, rather than page numbers.

*Line 188 - please clarify whether “level-2” PPE continued to be used for aerosol generating procedures or in other circumstances after 1 April 2020*

Yes, we have clarified this by explaining the following in the Methods section, Infection prevention and control measures subsection: “Prior to 1 April 2020 (phase 1) use of “level-1” personal protective equipment (PPE; apron, gloves, a surgical face mask and optional eye protection) was recommended for contact with patients known or suspected to have COVID-19 with use of “level-2” PPE (gown, gloves, eye protection, FFP3/N99 mask) for aerosol generating procedures. From 1 April 2020 (phase 2), in addition to the above, universal level-1 PPE was used for all patients regardless of test results or clinical suspicion of COVID-19.”

*Results: Line 281 (paragraph ‘Transmission risk to patients’) - For the adjusted analyses, please also provide the unadjusted analyses and indicate the factors which have been adjusted for.*

The corresponding unadjusted analyses are presented in the supplementary materials. All variables included in the adjusted analysis are listed in Table 3. We have provided this explanation in the Table 3 caption as follows: “Predictors of SARS-CoV-2 infection in admitted patients during their hospital stay from multivariable logistic regression results. All independent variables used in the multivariable regression model are listed in the table. The corresponding univariable analysis is presented in supplementary S2 Table S2.1.”

*Results: The OR results presented in Table 3 & 4 are presented in the main text as aOR - please clarify.*

We have changed the column headings in Table 3 and 4 to aOR to align with the main text.

*Results: Line 324 (paragraph ‘Transmission risk to healthcare workers’) - For the adjusted analyses, please also provide the unadjusted analyses and indicate the factors which have been adjusted for.*

The corresponding unadjusted analyses are presented in the supplementary materials. All variables included in the adjusted analysis are listed in Table 4. We have provided this explanation in the Table 4 caption as follows: “Predictors of SARS-CoV-2 infection in admitted patients during their hospital stay from multivariable logistic regression results. All independent variables used in the multivariable regression model are listed in the table. The corresponding univariable analysis is presented in supplementary S2 Table S2.2.”

*Results: Please clarify what the different phases (1-3) are in the main text of your results.*

The explanations for phases 1-3 are included in the Methods section, Infection prevention and control measures subsection.

*Results: Where OR / aOR are presented, please specify the comparison group.*

In tables 3 and 4, we have indicated a value of 1 in the cells for aOR for the comparison groups for categorical variables. In the main text, we have indicated the comparison groups by text for categorical variables.

*Discussion: Please present and organize the Discussion as follows: a short, clear summary of the article's findings; what the study adds to existing research and where and why the results may differ from previous research; strengths and limitations of the study; implications and next steps for research, clinical practice, and/or public policy; one-paragraph conclusion.*

We have reorganised and expanded the Discussion section as requested.

*Figures: Please consider avoiding the use of red and green in order to make your figure more accessible to those with colour blindness.*

We have changed the colors of the red and green circles in figure 1 to a color blind friendly palette.

*Tables: Tables 3 & 4 - please provide both OR and aOR results. The OR results presented in Tables 3 & 4 are presented in the main text as aOR - please clarify. Please specify the variables controlled for in the table legends.*

We have changed the column headings in Tables 3 and 4 from OR to aOR. The corresponding unadjusted analyses are presented in the supplementary materials. All variables included in the adjusted analysis are listed in Tables 3 and 4. We have provided this explanation in the Tables 3 and 4 captions as follows: “Predictors of SARS-CoV-2 infection in admitted patients during their hospital stay from multivariable logistic regression results. All independent variables used in the multivariable regression model are listed in the table. The corresponding univariable analysis is presented in supplementary S2 Table S2.1/S2.2.”

*Tables: When a p value is given, please specify the statistical test used to determine it in the respective figure legend*

We have included ‘p-values were calculated with the Wald test’ in the legends of Tables 3 and 4.

*Supplementary files: Please provide titles and legends for each individual table and figure in the Supporting Information.*

We have included all the titles and legends for each individual table and figure in the Supporting Information.

*Supplementary files: Tables S1 & S2 - please provide both OR and aOR results. Please specify the variables controlled for in the table legends. When a p value is given, please specify the statistical test used to determine it in the respective figure legend*

The corresponding adjusted regression results are presented in the main text (Tables 3 and 4). All variables included in the adjusted analysis are listed in Tables 3 and 4. We have provided this explanation in the Tables 3 and 4 captions as follows: Predictors of SARS-CoV-2 infection in admitted patients during their hospital stay from multivariable logistic regression results. All independent variables used in the multivariable regression model are listed in the table. The corresponding univariable analysis is presented in supplementary S2 Table S2.1/S2.2. We have included ‘p-values were calculated with the Wald test’ in the legends of Tables S1 and S2.

*Supplementary files: Please rename your supplementary files according to PLOS Medicine's style - Figure S1, Table S1 (rather than using subsections) and please ensure that all supplementary files are referred to in the main text of your manuscript e.g. Table S1 is not referred to*

We have renamed the supplementary files as requested, and ensured all tables and figures are referred to in the main text.

- **Comments from the reviewers - Reviewer #1**

*Thank you for inviting me to review this manuscript. Mo and colleagues analyzed patient and healthcare worker (HCW) data from 4 UK hospitals during their first COVID-19 surge (Jan-Oct 2020) in order to identify associations between various clinical factors and hospital-onset SARS-CoV-2 infections in patients and HCWs. The authors used a series of statistical models to identify the following primary findings: 1) for hospitalized patients, spending time in the same ward as another patient with hospital-acquired SARS-CoV-2 was associated with a much higher risk of infection compared to being on the same ward as a patient with community-acquired SARS-CoV-2 or being exposed to an infectious HCW; 2) for HCWs, exposures to an infectious patient with hospital-acquired SARS-CoV-2 or another infectious HCW were associated with higher infection risk than exposure to a patient with community-acquired infection. Overall, this paper has a wealth of data and addresses an important topic in trying to better understand the factors that drive nosocomial infection. However, I have some concerns about their methods and assumptions.*

*Major Comments:*

*1. An underlying assumption seems to be that most or all HCW SARS-CoV-2 infections were acquired in the hospital, either from patients or other HCWs. However, it is quite clear now that many, if not the majority, of HCW infections are acquired in the community rather than in the hospital. Put another way, I would expect the number of infected patients and HCWs on the same ward to be highly correlated with community incidence; at the same, high community incidence will translate into a higher risk of HCWs acquiring infection outside of the hospital. Unless I am missing something, I do not see any attempt to account for household or non-hospital exposures and to adjust for community incidence rates.*

Thank you for this important comment. We have now included infection pressure from the community as explained earlier.

*2. A major weakness is that no genetic sequencing was done to confirm any of the presumed transmission pathways. As above, it is entirely plausible if not likely that many HCWs were acquired outside the hospital. At the least, this needs to be acknowledged, and the language around in-hospital "transmissions" needs to be substantially softened.*

We have included this as a limitation in the Discussion section as follows: “Lastly, we did not consider the genomic sequences of the SARS-CoV-2 viruses to confirm the transmission pathways. A proportion of these infections in the HCW could potentially be introduced from the community. We attempted to address this by using admission rate of community-acquired cases to extrapolate community infection pressure, which overcomes the issue of low community testing in the initial phase of the pandemic.”

*3. Please describe the room set-up in the hospitals, as this is highly relevant when making the claim that patients infected other patients. In particular, are all rooms single hospital rooms or are they shared rooms? Were patients with suspected or confirmed COVID-19 placed in airborne infection isolation rooms or simply into standard rooms? Do patients congregate in shared areas? If patients are not in shared rooms, I think a more likely explanation for the association between hospital-onset infections with new infections is spread via intermediary HCWs.*

We have added a paragraph in the Methods section, Infection prevention and control measures subsection: “The hospitals consist of a mix of side-rooms and multi-occupancy bays of two to four individuals. Ventilation in these wards were largely via natural ventilation with windows. Known SARS-CoV-2 infected patients were cohorted into standard rooms. There were no airborne isolation rooms. The hospitals’ infection prevention and control strategies were implemented following the UK national guidelines. Since the first cases of COVID-19, HCW showing compatible symptoms were required to immediately isolate and obtain a PCR test. Patients were encouraged to wear masks at all times, especially if leaving their immediate bed area for an investigation. Visitors were generally not permitted during the pandemic period.”

*4. Were PCR cycle threshold values available at all, and if so was there any association with the risk of transmission? High viral loads have been shown to be highly correlated with transmission risk in other studies.*

We agree with the reviewer that this is an interesting idea. This would require a separate study which models the change in cycle threshold values with viral load over time. We proposed in the Discussion section that this can be further investigated with the data augmentation framework: “Further analysis using data augmentation methods, incorporating the PCR cycle threshold values, may potentially overcome this to produce estimates that better account for different sources of uncertainty.”

*5. Was there any consideration that positive PCR tests with high Ct values in later months of the study (when much of the population would have been infected) might represent residual RNA from prior infections rather than active, contagious infections? Recent data suggest that a large fraction of positive PCR tests in hospitalized patients after the first surge represent residual RNA from prior infections.*

We share this concern with the reviewer. Hence, we only considered the first positive PCR tests and censored subsequent observation days from these individuals after the first positive PCR tests. Also, as we state in the paper, most patients with apparent hospital-acquired infections had initial negative PCR tests while in hospital prior to their positive result “One-hundred and two out of the 130 patients had at least one negative PCR test during day 1-5 of their hospitalisation (79%).” This suggests that residual RNA form infections prior to hospital admission is unlikely to be a major factor here.

*6. I'm a bit concerned about the fact that 43% of HCW infections were identified based on serology and memory of their symptoms. How reliable is this?*

We agree with the reviewer that this is potentially associated with recall bias. We have added this in the Discussion as a limitation: “In HCW infections identified with serology, self-reported symptom onset dates may potentially suffer from recall bias.”

*7. The testing criteria for HCWs appeared to have been new symptoms, as well elective asymptomatic q2 week testing starting in late April 2020. Was there any requirement or recommendation for staff to get tested after unprotected exposure to patients or staff with COVID-19?*

Yes, this was implemented from June onwards and was explained in the Methods section, Infection prevention and control measures subsection: “Further measures were implemented subsequently from June onwards, which include universal masking and social distancing amongst staff, contact tracing and isolation of exposed patients and HCW, establishment of COVID-19 dedicated areas, improved triage and recognition of atypical symptoms in elderly patients.”

- **Comments from the reviewers - Reviewer #2**

*This paper uses routine data to examine infection risk in hospitals in Oxfordshire. I was asked for a statistical report and I interpret that to include all aspects of the design and conduct of the study.*

*Points of detail*

*Page 7 It is not clear to me what the force of `. . . who were on the same wards . . . ' is. Does this mean that they had to be on the same ward for their whole stay? But that seems to contradict the inclusion of location as time-varying later on this page. Table 2 seems to suggest that staff were only included if they served only one ward. If this is true is that not a substantial limitation as many categories of staff service multiple locations and even multiple hospitals?*

Thank you for raising this query. To improve clarity, we have added the following to the methods section, study cohort subsection: “Data were analysed taking individual infection outcomes for each person day at risk of infection as the outcome data. Thus the dependent variable was the binary outcome coded as 1 if the individual at risk became infected on that day and coded as 0 otherwise. Independent variables were classified as time-fixed and time-varying variables. Time-fixed variables included age at admission, sex and ethnicity routinely collected in hospital records. Time-varying variables included patients’ ward and hospital location, and the number of other patients and HCW known to be infected (and likely infectious) present on the same ward while a patient was at risk of becoming infected with SARS-CoV-2. Hospital HCW and patients who were on the same wards on the same day were included in the analysis.”

*Page 9 The definitions of community-acquired and hospital-acquired do not seem to be together exhaustive. If this is true what happened to the rest and is that a limitation?*

We agree with the reviewer that this is a limitation. We acknowledge this in the Discussion section: A key challenge in this analysis is that the times of infection are unknown. This has led to the adoption of various arbitrary cut-offs on length of stay prior to infection to define nosocomial infection. Further analysis using data augmentation methods may potentially overcome this to produce estimates that better account for different sources of uncertainty.

*Page 11 I think it would be usual to specify the risk factors which were going to be included in the models here. I know we can find out in Table 3 but that raises the question of whether all of the ones reported there were pre-specified or selected by some data-driven procedure. I*

*assume there was in fact no selection based on Tables S1 and S2 but it would be good to have it confirmed explicitly.*

Thank you for this comment. We have provided clarifications in the methods section that: “Independent variables in these regressions were chosen to describe the demographics of the individuals, setting of the hospital wards and infection pressures from patients and healthcare workers. The same set of variables were used in both univariable and multivariable logistic regression models.” The assumption that there was no selection is correct.

*Page 12 I am afraid I do not understand what relevance a re-admission has. If, as page 8 informs us, they can only be infected once then how does re-admission come into the picture?*

The purpose of highlighting the number of admissions in each patient group is mainly to describe the characteristics of the patients group classified as having nosocomial SARS-CoV-2 infections, which suggests that this group was likely to have chronic illnesses which required readmissions.

*Page 13 Collapsing together all other ethnic groups lacks a clear justification. If we take examples of heritages represented in the UK like Nigeria, Bangladesh, or Hong Kong, is there any reason to suppose they are all alike and different from white people? Table 3 even collapses*

*Other into non-White. I know the media and social statistics regularly collapse all non-White but that does not make it make scientific sense.*

We agree with the reviewer on this point. In our analysis, which classified the patient cohort into various groups, i.e., community-acquired, nosocomial acquired (with assumed incubation period of 3, 5, and 7 days), the number of patients in each non-White ethnic group was small. Hence, we decided to collapse the non-White ethnic groups into one.

*Page 17, Table 3 This might be better in landscape so the confidence intervals did not span rows. It is already broken over two pages so that is not an issue. The same applies to Table 4.*

Thank you for this suggestion. We will take note of this in the final formatting of the manuscript if it is accepted.

*Page 18 I am unconvinced that being multiplicative is a disadvantage of logistic models, that is just what they do, it is a feature.*

We agree with the reviewer. To clarify, we have expanded on this in the Results section, Transmission risk to patients subsection: “A multiplicative feature of the logistic regression model is that it assumed the effect of each additional infectious patient or HCW as multiplicative. To improve interpretability and applicability of the estimates in a clinical setting, we used a statistical model that allows each extra infectious individual to increase the probability of infection in an additive way (a generalised mixed model with an identity link).”

*References I know this is very picky but some of these have names which lack their appropriate initial capitals.*

Thank you for this comment. We have corrected these errors.

*Figure 3 This suffers from over-printing in the upper right panel. It might be worth experimenting with eliminating the fill colour.*

Thank you for your suggestion. We have increased the transparency of the fill colours to prevent over-printing. We experimented with using lines instead of fill colors for the standard errors. However, this produced multiple lines which may be confusing for the readers.

*Points of more substance: Additive versus multiplicative*

*The abstract concentrates on the additive model but in the results it is presented as an alternative rather than as the primary model. Absolute risk as opposed to relative has many advantages, I am not objecting to the choice in the abstract, but it seems inconsistent to make the multiplicative the main focus in the results.*

We agree with the reviewer that how we phrased the additive model as ‘alternative’ could be confusing. We have removed the word ‘alternative’ when it was used in conjunction with the additive model in the Results section.

*What are UK hospitals like?*

*If this is going to be useful to readers in other countries do they need to know more about the usual layout of hospital wards in the UK or in these hospitals specifically? I fortunately have limited knowledge of this but my impression is that four- and six-bedded bays are common here so results might be different in countries which have single rooms or Nightingale wards.*

We have added a paragraph in the Methods section, Infection prevention and control measures subsection: “The hospitals consist of a mix of side-rooms and multi-occupancy bays of two to four individuals. Ventilation in these wards were largely via natural ventilation with windows. Known SARS-CoV-2 infected patients were cohorted into standard rooms. There were no airbone isolation rooms. The hospitals’ infection prevention and control strategy were implemented following the UK national guidelines. Since the first cases of COVID-19, HCWs showing compatible symptoms were required to immediately isolate and obtain a PCR test. Patients were encouraged to wear masks at all times, especially if leaving their immediate bed area for an investigation. Visitors were generally not permitted during the pandemic period.”

*Summary*

*Mostly points for clarification.*

*Michael Dewey*

- **Comments from the reviewers - Reviewer #3**

*Dr. Mo and colleagues present very interesting data quantifying the frequency of potential hospital-acquired SARS-CoV-2 and quantifying the differential risk of infection via potential exposure to community-acquired vs hospital-acquired vs healthcare worker cases. These data are valuable because while nosocomial spread of SARS-CoV-2 has been well reported it has not been well characterized in terms of incidence and risk factors. Strengths of the study include drawing data from multiple hospitals, detailed daily level analyses, and differentiation of different kinds of exposures. Weaknesses include the indirectness of attribution of infections - these are imputed from associations rather than confirmed through detailed case-by-case analyses or sequencing. Nonetheless, the results are informative and welcome.*

*1. Text (line 234) says 97/920 patients tested positive for SARS-CoV-2 on day 7 or later but Table 1 says 120/920 patients tested positive assuming incubation period of 7 days. Please clarify this discrepancy. Does the latter include patients who tested positive post-discharge? If so, shouldn't the denominator be larger?*

Thank you for your comment. The denominator, 920 patients, refers to all patients who had a positive SARS-CoV-2 PCR test during the study period. This should remain the same regardless of the definitions used to classify the community- or hospital-acquired infections. Ninety-seven patients in the first statistic above were indeed patients who had continuous hospitalisation for seven days or more, and 120 patients were those who were admitted on the 7th day prior to their first positive SARS-CoV-2 PCR test. To be more clear, we have edited the explanation for the 97/920 patients (Results section Patient characteristics subsection) as follows: Out of these, 571 patients had their first positive PCR tests while hospitalised (62%), and 97 were continuously admitted for at least 7 days (11%).

*2. Do the authors have access to cycle thresholds? Comparing these values across community onset, hospital-acquired, and HCW infections could support their evaluations of infectiousness of these different groups*

We agree with the reviewer that this is an interesting idea. This would require a separate study which models the change in cycle threshold values with viral load over time. We proposed in the Discussion section that this can be further investigated with the data augmentation framework: “Further analysis using data augmentation methods, incorporating the PCR cycle threshold values, may potentially overcome this to produce estimates that better account for different sources of uncertainty.”

*3. Can the authors say more about their baseline infection prevention infrastructure. Are patients in shared rooms or private rooms? What are the ventilation standards for hospital rooms? Are patients required to wear masks in the hospital? Were visitors permitted and if so were they tested?*

We have added the following in the Methods section, Infection prevention and control measures subsection: “The hospitals consist of a mix of side-rooms and multi-occupancy bays of two to four individuals. Ventilation in these wards were largely via natural ventilation with windows. Known SARS-CoV-2 infected patients were cohorted into standard rooms. There were no airborne isolation rooms. The hospitals’ infection prevention and control strategy were implemented following the UK national guidelines. Since the first cases of COVID-19, HCWs showing compatible symptoms were required to immediately isolate and obtain a PCR test. Patients were encouraged to wear masks at all times, especially if leaving their immediate bed area for an investigation. Visitors were generally not permitted during the pandemic period.”

*4. Did the authors evaluate differences in risk in patients who shared a room with someone with hospital-acquired SARS-CoV-2 versus those who were simply on the same ward?*

Thank you for this comment. We agree that this is an important consideration, though details of patients’ locations within wards on each day of stay were not available to us, so an analysis at this level of granularity was not possible. However, we accommodated for the differences in the ward set-up by using mixed-effects models, and allowed the daily probability of infection (intercepts and slopes in the models) to vary by wards. The final estimates presented are the mean and 95% credible intervals of the mean estimates obtained for each ward. To explain this, we have included the following in the Methods section: Because the ward set-ups such as number of beds, isolation facilities, staff to patient ratio and infection prevention and control measures vary, we allowed the daily probability of infection (intercepts and slopes in the models) to vary by ward. The final estimates presented subsequently are the mean and 95% credible intervals of the mean estimates obtained for each ward.

*5. Were positive healthcare workers and patients interviewed to try to determine their particular exposures? Any patterns evident in interview-based exposure analyses?*

We agree with the reviewer that this would be very helpful. Given the challenges especially during the COVID-19 pandemic, we did not perform the interviews.

- **Comments from the reviewers - Reviewer #4**

*Mo Y et al. report their results regarding their investigations into transmission pathways of SARS-CoV-2 among hospitalized patients and healthcare workers at four teaching hospitals in Oxfordshire. They found exposure to patients with hospital-acquired-SARS-CoV-2 to pose a substantial risk of subsequent infection leading to the conclusion that infection control measures to protect both patients and healthcare workers need to be optimized.*

*The topic is important and of interest to the readers of PLOS Medicine. Yet, I have the following concerns:*

*Among healthcare workers testing positive for SARS-CoV-2, 43% of the diagnosis were based on serology - how was the time of infection determined in these cases (given the intrinsic limitations of drawing conclusions regarding time of infection based on serology)*

Thank you for raising this point. Healthcare worker infections were identified “using PCR results from symptomatic and asymptomatic testing at the hospital. Symptomatic testing was offered to staff from 27 March 2020 onwards and staff could participate in a voluntary asymptomatic screening programme from 23 April 2020 onwards, offering testing up to once every two weeks. Additionally, probable infections prior to widespread availability of testing were identified in staff without a positive PCR result, but who were either anti-nucleocapsid or anti-spike IgG antibody positive and recalled a date of onset of symptoms consistent with COVID-19. These symptoms were the presence of fever and new persistent cough, or anosmia or loss of taste.” This has been explained in the Methods section, Study cohort subsection.

*One of the main conclusions is that presence of patients with hospital-acquired SARS-CoV-2 was associated with the highest risk of acquisition in susceptible patients - given the nature of the data it is unclear how cause and effect can be distinguished here - how did the authors distinguish between all patients on a specific ward having been infected by one common index from ongoing exposure to subsequently infected patients? The current statement suggests that presence of patients with healthcare-associated SARS-CoV-2 infection puts other patients at risk for subsequent infection, yet all patients on a specific ward may have been infected by the same common source (resulting in differing times to detection, based on different timing of exposure and incubation periods).*

Thank you for the comment. To be clear on the analysis method, we have expanded the Methods section, Study cohort subsection as follows: “Data were analysed using each patient day as a data point. This consists of a binary dependent variable, i.e., if an individual becomes infected on the day or not, and independent variables which were classified as time-fixed and time-varying variables. Time-fixed variables included age at admission, sex and ethnicity routinely collected in hospital records. Time-varying variables included patients’ ward and hospital location, and the number of other patients and HCW known to be infected (and likely infectious) present on the same ward while a patient was at risk of becoming infected with SARS-CoV-2. Hospital HCW and patients who were on the same wards on the same day were included in the analysis.”

Analysing the data on a patient day basis differentiates the two scenarios described by the reviewer: “all patients on a specific ward having been infected by one common index from ongoing exposure to subsequently infected patients”. For example, a single infectious patient who stayed in the ward for 10 days while he was infectious would have contributed to the infection pressure for 10 data points for all other susceptible patients who were present in the ward on those 10 days. This is in contrast to having 10 other infectious patients who stayed in the same ward for 1 day, which would reflect in the data as 10 times the infection pressure (of the previous case) for 1 data point for all other susceptible patients present on that day. The presence of patients with healthcare-associated SARS-CoV-2 infection puts other patients at risk for subsequent infection is in our opinion the most parsimonious explanation, but of course we cannot completely rule out other possible explanations for the associations seen.

*Presumably, patients with community-onset SARS-CoV-2 infection, were identified early during their course of hospitalization and placed under isolation precautions - it therefore seems clear that the risk of subsequent transmission should be reduced. The analyses does not seem to account for this and suggests an assumption of an equal risk which does not seem justifiable.*

We agree with the reviewer that this is indeed likely to be true from the analysis results. Although we did not explicitly account for the reduced infectiousness from infection prevention and control measures, by considering the community- and hospital-acquired infection as separate drivers of nosocomial SARS-CoV-2 infections, we have quantified the probabilities of being infected from exposure to either groups separately. In our discussion ,we raise the point suggested by the reviewer: “The relatively low risk of transmission associated with patients with suspected community-acquired COVID-19 suggests that for these patients the peak of their infectivity may have passed such that existing infection prevention and control policies including universal PPE, prompt testing and isolation of suspected or known cases are sufficient to mitigate most of the remaining infectiousness.”

*How was cause and effect distinguished when analyzing transmission risks to healthcare workers? It is extremely challenging to analyze direction of transmission (i.e. from infected healthcare worker to patient or vice versa) - the data suggests that direction of transmission was clear in this dataset - how was it determined?*

We agree with the reviewer that determining the direction of transmission is indeed challenging, and had we not had high resolution temporal data on timings of ward-level exposures is not something we would have attempted. We attempted to do so by analysing the data using each patient day as a data point, and differentiating the groups of potentially infectious individuals present on the same day and wards. For example, consider a patient who became PCR positive on day 20 of admission (likely to have been infected on day 15 of admission based given an assumed incubation period of 5 days). The data point for this patient on day 15 of admission would have an outcome value of 1 and the independent variables would consist of the number of other infectious patients and healthcare workers on the same day, i.e., if A is infected first, B is exposed to A and develops symptoms later, it is more likely that A infected B than the other way around.

In addition, throughout the manuscript we described the transmission pathways as ‘associations’ rather than ‘causal’ in order not to overstate our findings.

*It is unclear, why the dates on symptom onset for patients were not available - it is comment practice to document such information in medical records. As this data would enhance the quality of the study substantially, the authors should try to collect this information or explain in more detail why it is missing.*

We agree that this information is valuable for our analysis. Our team of co-authors (included in the supplementary S7) performed chart review on 245 inpatients. We found that the mean interval between symptom onset and swab for PCR-testing was one day (interquartile range 1-3). This underpins our assumption to determine the symptom onset dates relative to the PCR tests. We did not persist in completing the chart review for all the participants in our study due to limited resources and tightening infection control measures during the pandemic.

Thank you and we look forward to further correspondence.

Yours sincerely,

Mo Yin

MRCP
