# Supplementary material for: Transmission of community- and hospital-acquired SARS-CoV-2 in hospital settings in the UK: A cohort study
Source: PLoS Med. 2021 Oct 12;18(10):e1003816. doi: 10.1371/journal.pmed.1003816 (PMC8509983; doi:10.1371/journal.pmed.1003816)
Supplement: Attachment — Submitted filename: covidtransmission_plosreviewreply.docx [file pmed.1003816.s012.docx]

Professor Raffaella Bosurgi

*Editor-in-Chief*

*PLoS Medicine*

3 September 2021

Dear Professor Bosurgi,

**Re: Manuscript submission: “Transmission of community- and hospital-acquired SARS-CoV-2 in hospital settings in the UK: a cohort study” for PLoS Medicine**

Thank you very much for the reviewers’ and the editorial board’s comments, and for accepting our manuscript. Please find the accompanying revised manuscript for your review. Our responses to the comments are as follows.

- Title

*Please add ‘in the UK’ to your title. We suggest “Transmission of community- and hospital-acquired SARS-CoV-2 in hospital settings in the UK: a cohort study”* 

We have changed the title as suggested.

- Abstract

*Please structure your abstract using the PLOS Medicine headings (Background, Methods and Findings, Conclusions).*

We have structured the abstract according to the recommendations.

*The final sentence of your Abstract Background should clearly state the study question e.g. “The aim of our study was to…”* 

We have added to the end of the abstract background: “The main aim of our study was to quantify the relative importance of different transmission pathways of SARS-CoV-2 in the hospital setting.”

*Abstract Methods and Findings: Line 54 - please specify the incubation periods used to define community- or hospital-acquired cases*

We have specified the incubation period by changing the sentence to: “Cases were classified as community- or hospital-acquired using likely incubation periods of three to seven days.”

*Abstract Methods and Findings: Please report percentages to at least one decimal place.*

We have reported the percentages to at least one decimal place.

*Abstract Methods and Findings: Please provide mean  +/- SD for age*

We have reported age with the standard deviation.

*Abstract Methods and Findings: Line 61 - please report results on infections per 1000 susceptible patients per day to one decimal place*

We have reported age with the standard deviation.

*Abstract Methods and Findings: Line 62 - please add ‘per day’*

We have added ‘per day’ and changed the sentence to: “For susceptible patients, one day in the same ward with another patient with hospital-acquired SARS-CoV-2 was associated with an additional 7.5 infections per 1000 susceptible patients (95% Credible Interval (CrI) 5.5 to 9.5/1000 susceptible patients/day) per day.”.

*Abstract Methods and Findings: Line 71 - Please move the final sentence earlier within your Abstract Methods and Findings*

We have moved the final sentence earlier in the paragraph.

Abstract *Conclusions: line 75 - please be careful to avoid inadvertent use of causative language - please revise “pose a substantial infection risk” to speak instead of associations*

We have changed the sentence to: “In this study, we observed that exposure to patients with hospital-acquired SARS-CoV-2 is associated with a substantial infection risk to both healthcare workers and other hospitalised patients”.

- Author summary

*Please revise ‘nosocomial’ on line 89, which may not be a familiar term to a non-technical audience, and temper inadvertent causative language on line 110 to speak instead of associations.*

We have changed this to “hospital-acquired”.

*In the final bullet point of ‘What Do These Findings Mean?’, please describe the main limitations of the study in non-technical  language.*

We have added the following as a bullet point: “The main limitations were that the symptom onset dates and healthcare worker absence days were not available, which may affect the estimation of the transmission pathways.”

- Methods

*Please state early in the Methods section that your study did not have a prospective protocol or analysis plan, alongside your statement that ‘Analyses were data-driven’. We suggest adding this in the paragraph starting on line 185.* 

We added the following sentence to the suggested paragraph: “The study did not have a prospective protocol or analysis plan and the analyses were data-driven.”

- Results

*Please report percentages to at least one decimal place.*

We have reported percentages to one decimal place.

- Discussion

*Line 453 - please revise to ‘ associated with more modest increases’*

We have changed the sentence to: “In contrast, exposure to patients who had acquired SARS-CoV-2 in the community appeared to be associated with more modest increases in the daily risk of infection for both healthcare staff and the other patients.”

*Line 526 - please be careful to avoid inadvertent use of causative language - please revise “pose a high risk” to speak instead of associations*

We have changed the sentence to: “In conclusion, our data provide strong evidence that newly infected patients are associated with a high risk of onward transmission to patients and HCW in hospital.”

- Figures:

*Figure 1 legend - sentence ending ‘consistent with COVID-19’ appears incomplete*

We have changed the sentence to: “We assumed that PCR tests were performed one day after developing symptoms which were consistent with COVID-19.”

*Figures 4 & 5 - please clarify what is represented by the central dot within each horizontal bar*

We have added to the figure captions: “The black crosses in the centre of each bar represent the median of the estimates.”

*Figure S7  - please amend typographical error in x axis label*

We have corrected the error in the x-axis label in Figure S7.

- Tables

*Tables 3 & 4 - please add sentences beginning “all independent variables...' and ‘The corresponding univariable’ as footnotes. Please make it clear in the table footnote that a value of 1 in the cells for aOR represents the comparison group for categorical variables (you may wish to denote this with an additional number in superscript)*

We have moved sentences beginning “all independent variables...' and ‘The corresponding univariable’ as footnotes. We have also added an additional number in the superscript to explain that a value of 1 in the cells for aOR represents the comparison group for categorical variables.

- Supplementary files:

*Please amend references contained in Figure S6 to adhere to PLOS Medicine style* [*https://imsva91-ctp.trendmicro.com:443/wis/clicktime/v1/query?url=https%3a%2f%2fjournals.plos.org%2fplosmedicine%2fs%2fsubmission%2dguidelines%23loc%2dreferences&umid=662AD589-CB16-FE05-9277-5ABBEAF9AFFF&auth=6e3fe59570831a389716849e93b5d483c90c3fe4-6230ae3e61e01af1d6dc1ff6c40e11c348e3fcc9*](https://imsva91-ctp.trendmicro.com:443/wis/clicktime/v1/query?url=https%3a%2f%2fjournals.plos.org%2fplosmedicine%2fs%2fsubmission%2dguidelines%23loc%2dreferences&umid=662AD589-CB16-FE05-9277-5ABBEAF9AFFF&auth=6e3fe59570831a389716849e93b5d483c90c3fe4-6230ae3e61e01af1d6dc1ff6c40e11c348e3fcc9)

We have amended the references in S6 to adhere to PLOS Medicine.

Thank you and we look forward to further correspondence.

Yours sincerely,

Mo Yin

MRCP
